# Assessing National Health Research System in a resource-limited setting: Insights from Indonesia

**DOI:** 10.1371/journal.pone.0350393

**Published:** 2026-06-04

**Authors:** Tommy Dharmawan, Deandra Ardya Sutoyo, Fona Qorina, Nico Gamalliel, Mohammad Kurniawan, Dian Kusuma, Ahmad Fuady

**Affiliations:** 1 Evidence-based Health Policy Centre, Indonesian Medical Education and Research Institute, Faculty of Medicine, Universitas Indonesia, Jakarta, Indonesia; 2 Department of Surgery, Faculty of Medicine, Universitas Indonesia, Jakarta, Indonesia; 3 Department of Neurology, Faculty of Medicine, Universitas Indonesia, Jakarta, Indonesia; 4 Department of Public Health and Epidemiology, College of Medicine and Health Sciences, Khalifa University of Science and Technology, Abu Dhabi, United Arab Emirates; 5 Department of Community Medicine, Faculty of Medicine, Universitas Indonesia, Jakarta, Indonesia; Airlangga University Faculty of Medicine: Universitas Airlangga Fakultas Kedokteran, INDONESIA

## Abstract

High-quality health research is essential for evidence-based policymaking and health system strengthening. Indonesia has undergone major reforms in its health research governance. However, the country’s National Health Research System (NHRS) remains insufficiently mapped. This study aimed to assess the current status of Indonesia’s NHRS using the WHO framework, which includes four domains: governance, financing, creating and sustaining resources, and producing and using research. A qualitative approach was employed, combining an expert panel discussion and key informant interviews with 13 participants consisting of national and subnational stakeholders from government agencies, research organisations, and universities from May to December 2024. Data were analysed using the Framework Method guided by the NHRS framework and supplemented by the WHO Questionnaire on Country Resources for Health Research. This study found that, despite progress in Indonesia’s NHRS, weak governance, particularly poor coordination between national and subnational levels, remains the main challenge. Overall, the system is still fragmented, even though formal structures and institutions are in place. Governance is characterised by a highly centralised, top-down approach to agenda setting, with limited engagement of local institutions. Research financing is mainly programmatic and proposal-based, aligned with national priorities, but is constrained by limited sustainable domestic funding and unequal access to funding. In terms of resources, advanced research infrastructure and growing international collaboration indicate national commitment to health research; however, mismatches between infrastructure and human resource capacity persist. Finally, although research production and utilisation focus on measurable outputs and are supported by mechanisms for evidence use, effective pathways for translating research into policy and practice remain limited. Improving Indonesia’s health research system calls for an integrated national research agenda, well-defined institutional roles, and streamlined coordination between national and regional levels.

## Introduction

High-quality health research is one of the keys to strengthening health systems, advancing equity, and informing effective policies [1]. A national health research system (NHRS) is defined as “the people, institutions, and activities whose primary purpose is to generate knowledge and promote its utilisation to improve health and health equity” [2]. Based on the World Health Organization (WHO), NHRS consists of four core domains, which are governance, financing, creating and sustaining resources, and producing and utilising research [2]. Strengthening these domains collectively fosters an enabling environment for evidence generation and uptake, thereby contributing to improved broader public health outcomes and broader national development goals [2^,^^3^].

Despite its importance, the NHRS remains underexplored in low- and middle-income countries (LMICs). LMICs bear 92% of the global disease burden yet remain under-represented in health research production and agenda-setting [4,5]. Research priority setting in LMICs is often fragmented, donor-driven, and poorly implemented, with less than half of initiatives aligned with national strategies [2]. Moreover, only 2% of global health aid from 2000 to 2014 was allocated to research, mostly from a handful of donors and largely targeted at global rather than country-specific needs [6–8]. This donor dominance contributes to weak national ownership in research, misaligned priorities, and limited political commitment to invest in domestic research systems [4].

Even when local researchers generate relevant evidence, systemic inequities persist. LMICs researchers remain under-represented in global publications. For instance, in the field of global surgery, 51% of authors were affiliated exclusively with high-income countries (HICs), and over two-thirds of first and last authors came from HICs institutions [9]. This imbalance may reflect not only systemic publication bias but also the limited research capacity in many LMICs, underscoring the urgent need for sustainable investment in capacity strengthening, governance reform, and national research ownership [5]. Based on the WHO’s report on gross domestic research and development expenditure on health (health GERD), as a percentage of Gross Domestic Product (GDP), upper-middle, low, and lower-middle-income countries spend less than 0.03% of their GDP on health. In contrast, HICs overall spend on health GERD 0.27% of their GDP, and the proportion even reaches 0.93% in Denmark [10].

Indonesia presents as a strategically important case for examining NHRS performance in LMICs context. As an upper-middle-income and the fourth most populous country with more than 288 million inhabitants, Indonesia’s health system mirrors those in many LMICs, including low research funding, resource shortages, and weak knowledge-to-policy translation [11].

Indonesia has recently undergone a reformation of its national research governance structure. In 2020, the government merged multiple research agencies from different ministries, including health research agencies, into a single, central-level research entity: the National Research and Innovation Agency, or *Badan Riset dan Inovasi Nasional* (BRIN), directly responsible to the president [12]. While this reform aimed to consolidate existing research agencies under one coordinating agency, centralising functions previously embedded within different institutions carries its own risks and, such structural complexity may pose new challenges to the coherence and functionality of Indonesia’s NHRS [13]. Whether the reform has strengthened Indonesia’s NHRS remains unanswered.

The governance challenges coincide with epidemiological transitions from infectious to non-communicable diseases, compounded by population ageing, rapid urbanisation, and changing lifestyles. These shifts demand integrated and forward-looking research agendas capable of addressing both emerging and persistent health challenges. Assessing the current performance and strengthening efforts of NHRS in Indonesia is therefore important. The lessons learned from Indonesia’s NHRS context, especially with the latest reform of Indonesia’s National Research Agency, may help improve NHRS in other LMICs, particularly those considering similar governance reform. Such evidence is especially timely in LMICs settings where health demands are continuing to rise, yet resources are scarce.

Previous studies have examined the status of NHRS in other settings, such as the work of Kirigia and Wambebe (2015) [14] in the WHO African Region and Musango et al. in Mauritius [15]. However, similar studies about the assessment of NHRS in Asian contexts remain limited; data on Indonesia’s expenditure on health research and development is not even available in the WHO Report. Therefore, to fill this gap, this study aimed to explore and assess the current status of Indonesia’s NHRS. The findings are intended to inform future strategies for strengthening research governance, promoting evidence generation and evidence-informed policymaking, and improving the overall performance of the broader health system, with transferable lessons for NHRS reform in LMICs.

## Methods

### Study design and context

To assess the current status of Indonesia’s NHRS, a qualitative approach was used to explore stakeholders’ perspectives. In this study, “health research” was defined as any research activities that contribute to the strengthening of the health system, improving population health, and informing evidence-based health policy [14]. This includes biomedical, clinical, public health, and health systems research conducted by government institutions, universities, and research organisations at both national and subnational levels. The scope also encompasses research related to health policy, governance, financing, and innovation that supports the broader objectives of national health development.

Since 2020, Indonesia’s research ecosystem has been primarily managed by two key institutions: BRIN and the Ministry of Higher Education, Science and Technology *(Kementerian Pendidikan Tinggi, Sains, dan Teknologi*, Kemendikti-Saintek). BRIN is a centralised agency established through the merger of several previous research institutions. Previously, health-related research had been managed by the Health Research and Development Agency (*Badan Penelitian dan Pengembangan Kesehatan*, Balitbangkes) under the Ministry of Health (*Kementerian Kesehatan*, Kemkes), the Eijkman Institute, the Indonesian Institute of Sciences (*Lembaga Ilmu Pengetahuan Indonesia*, LIPI), and other agencies, while Kemendikti-Saintek oversees the research conducted by universities and colleges. Both BRIN and Kemendikti-Saintek operate directly under the president [16]. In addition to these two institutions, the Ministry of Religious Affairs (*Kementerian Agama,* Kemenag) coordinates religion-based universities and colleges, which also conduct research activities. On the funding side, domestic research funding increasingly comes from the Indonesia Endowment Fund for Education Agency (*Lembaga Pengelola Dana Pendidikan*, LPDP), which plays an expanding role in supporting national research and innovation.

### Participants’ selection and recruitment

Participants were selected using purposive sampling to ensure representation from key stakeholders involved in the governance and implementation of health research in Indonesia. Eligible participants were individuals with demonstrated experience in research management and policy at national, subnational, and institutional levels (Table 1). This included academic stakeholders holding at least a lecturer-level position (or equivalent), as well as policymakers occupying leadership roles (heads of units, divisions, or departments). To ensure a strategic and informed perspective, the study prioritised senior representatives with a minimum of five years of relevant experience in their respective fields.

**Table 1:** List of participants.

Method	Level	Institution
Expert panel	National	Health Development Policy Agency, Ministry of Health (BKPK-Kemkes) (N01)
		Ministry of Higher Education, Science and Technology (Kemendikti-Saintek) (N02)
		Health Research Organization, National Research and Innovation Agency (BRIN) (N03)
	Institutional representatives	Universities and research institutions (Western Indonesia) (W01, W02)
		Universities and research institutions (Central Indonesia) (C01, C02)
		Universities and research institutions (Eastern Indonesia) E01, E02, E03)
	Subnational	Regional/Subnational Research Organisation, BRIN (BRIDA) (N04)
Key informant interviews	National	Health Technology Assessment Division, Ministry of Health (HTA-Kemkes) (N05)
		Health Research Organization, National Research and Innovation Agency (BRIN) (N03)

To ensure geographic diversity, invitations were extended to central government agencies and academic institutions from Western, Central, and Eastern Indonesia. At the national level, four key policymaking institutions were represented: the Health Research Organisation of the BRIN, the Deputy for Regional Research and Innovation (*Badan Riset dan Inovasi Daerah*, BRIDA) under BRIN, the Health Development Policy Agency of the Ministry of Health (BKPK-Kemenkes), and the Kemendikti-Saintek.

From the institutional level, participants were drawn from universities and research institutes engaged in research planning and management. The academic cohort included two participants from Western Indonesia, two from Central Indonesia, and four from Eastern Indonesia. Most were affiliated with medical faculties or research centres, providing perspectives on institutional-level research management and subnational health research needs. These academicians were specifically selected for their leadership roles in organising and executing health research, particularly in relation to national research funding and programme alignment. Efforts were made to ensure diversity in institutional roles, gender, and subnational representation in line with purposive sampling goals.

The participant recruitment period was conducted from 20/05/2024–30/05/2024. The sample size was predetermined during the study design. All eligible key informants received formal invitations via e-mail, accompanied by an explanation of the study objectives. Follow-up communication by phone or text was conducted within two to three days to confirm participation.

### Data collection

Data were collected through an expert panel meeting and key informant interviews. The expert panel meeting was conducted on 31/05/2024 in a hybrid format, held in person at the Faculty of Medicine, Universitas Indonesia, and online via Zoom to enable broad participation and reduce logistical constraints. The hybrid approach is feasible and valid in qualitative policy research involving geographically dispersed participants [17].

Of the 13 stakeholders invited, 11 attended the expert panel meeting. Those who were unable to participate were subsequently invited for individual key informant interviews, which were conducted on 13/12/2024 and 16/12/2024. Additional interviews were also conducted with selected participants who were considered to have in-depth insights essential for achieving a comprehensive understanding of Indonesia’s NHRS. All participants provided written informed consent, and sessions were audio-recorded with permission. Data saturation was reached when no new themes or insights emerged during the final interviews.

The expert panel was facilitated by the principal investigator (TD) to discuss four main areas: (a) how institutions set health research priorities, (b) stakeholders involved in the process, (c) challenges in conducting and implementing research aligned with national priorities, and (d) critical enablers and barriers in strengthening Indonesia’s health research system. We conducted and transcribed verbatim the discussion and interviews in Bahasa Indonesia. The transcripts were securely stored and only accessible to the researchers.

### Data analysis

Transcripts were later imported into NVivo (version 12). Analysis was conducted using the Framework Method [18] and guided by the NHRS framework [19], consisting of four core domains: governance, financing, creating and sustaining resources, and producing and using research (**Table 2**). This framework served as the analytical foundation to understand the functions, dynamics, and coordination of Indonesia’s health research system. A combination of deductive and inductive analysis was applied. The Steps for Coding and Theorization (SCAT) method [20] was employed, as it is well-suited for small-to-medium qualitative datasets. Initial coding was independently conducted by two researchers (FQ and DA). Any discrepancies were resolved through discussions until consensus was achieved. Codes were then synthesised into subthemes and grouped into broader themes aligned with the NHRS framework.

**Table 2:** Domains of NHRS.

Domains	Definition
Governance	The policy and legal frameworks, along with institutional structures, that guide and regulate the NHRS. It includes setting ethical standards and ensuring ethical governance
Financing	Involves securing and managing funding for health research. It includes sourcing funds at subnational and national levels and contributing to broader funding schemes or programs.
Creating and sustaining resources	Focuses on building and maintaining both human and institutional capacities. It includes developing skills and competencies of health research personnel, strengthening institutions such as universities and research centres, and establishing the necessary infrastructure to support research activities.
Producing and utilising research	refers to the production (research projects/programmes, publications) and use (dissemination, communication, translation) of knowledge.

Data were triangulated across multiple sources. Documentary data were used to map the structural and institutional landscape of the NHRS, while interview data from expert panel discussions and key informant interviews provided contextual insights into how these structures function in practice. The integration of these data sources enabled both validation of documented evidence and the identification of gaps between formal arrangements and operational realities.

In addition, the analysis incorporated selected domains from the Questionnaire on Country Resources for Health Research [21], a tool developed by the WHO (S1 Appendix) and previously assessed in the WHO Africa Region [14,15]. Of the ten categories in the original questionnaire, we only assessed Indonesia’s NHRS using seven categories that are relevant and applicable to the Indonesian context: health research policy, legislation, strategic planning, coordination mechanisms, research programmes, research institutes, and financing and budgeting**.** Two researchers (FQ and DA) completed the questionnaire items based on publicly available data sources.

Representative quotes were identified and translated from Bahasa Indonesia into English, followed by back-translation to preserve meaning and contextual nuance. Finally, several team meetings were held as part of the triangulation process to refine themes according to the NHRS framework and validate interpretations. Inconsistencies in categorisation were discussed and reconciled.

### Ethics

Ethical clearance was obtained from the Health Research Ethics Committee, Faculty of Medicine, Universitas Indonesia, No. KET-658/UN2.F1/ETIK/PPM.00.02/2024. Written informed consent was obtained from all participants in the study.

## Results

A total of 13 participants contributed to the study. Of these, 11 participated in the expert panel discussion, and two participated in key informant interviews, one of whom was also a member of the expert panel. Data saturation was achieved through thematic saturation, as all domains of the NHRS framework were sufficiently explored and no new theme emerged from the data. At this point, additional data collection was deemed unlikely to generate further insights relevant to the study objectives.

### Current state of NHRS in Indonesia

**Fig 1:**
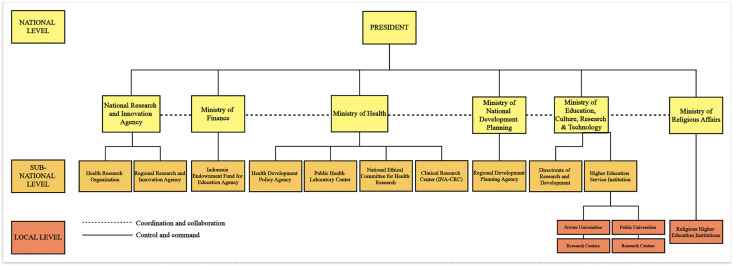
Current governance landscape of Indonesia’s health research ecosystem.

At the national level, BRIN serves as the central body coordinating research and innovation across all sectors, including health. BRIN oversees subnational research bodies, known as BRIDA, which function to link national research priorities with subnational implementation (Fig 1). Guided by its Strategic Plan 2022–2024 (BRIN Regulation No. 6 of 2023), one participant (N03) noted that *“BRIN performs a distinctive triple role: as a funder, implementer, and policy advisor—a governance model which is rarely observed internationally.”*.

The Ministry of Health primarily focuses on translating research into health policy and practice through its policy and ethics bodies, including BKPK-Kemkes and the National Ethical Committee for Health Research, as well as through clinical and public health research infrastructures such as the Indonesia Clinical Research Center (INA-CRC) and public health laboratories.

The Ministry of National Development Planning (*Kementerian Perencanaan Pembangunan Nasional,* Bappenas) aligns health research activities with the country’s medium and long-term development goals. Research within higher education institutions is governed by Kemendikti–Saintek, through the Directorate of Research and Development and the Higher Education Service Institutions, overseeing both public and private universities and their affiliated research centres. In parallel, Kemenag manages religious higher education institutions, such as Islamic universities, which also conduct research and contribute to the national health research ecosystem.

In terms of financing, health research in Indonesia is supported through two main funding streams. First, BRIN manages national competitive research funding through several schemes. These schemes are primarily funded by the Indonesia Endowment Fund for Education *(Lembaga Pengelola Dana Pendidikan*, LPDP), offer relatively larger budgets, and are open year-round, although they generally require the involvement of BRIN researchers. Second, BRIN also provides funding through internal schemes managed by individual Research Organizations and largely financed through the state budget *(Anggaran Pendapatan dan Belanja Negara*, APBN).

In the health sector, these function as seed funding with stricter eligibility criteria, requiring BRIN researchers to serve as principal investigators. In parallel, universities access research funding through Kemendikti–Saintek via institutional research offices *(Lembaga Penelitian dan Pengabdian kepada Masyarakat,* LPPM); however, these funds are comparatively limited. Overall, while BRIN, particularly through LPDP-funded schemes, constitutes the main source of national research funding, APBN-based allocations remain constrained and subject to annual budget negotiations.

At the subnational and local levels, most operational research is conducted by BRIDA, universities, and health research centres. In addition, independent research institutions and private-sector actors, including industry-based research units and contract research organisations, also contribute to research activities, particularly in applied and clinical research. The overview of Indonesia’s NHRS is presented in **Table 3**.

**Table 3:** Current situation and challenges of Indonesia’s NHRS.

Domain	Current Situation	Challenges
Governance	- Highly centralised at the agenda-setting level: top-down frameworks - Priority setting is based on the national medium- and long-term plan - Multiple research agencies with each institution’s research plan.	- Fragmented inter-institutional coordination - Limited local relevance and involvement in national priority setting
Financing	- Matching funds with national priorities - Programmatic and proposal-driven schemes - Strong reliance on external funding	- Constraint of sustainable domestic funding - Inequitable access to funding - Lack of pathways for domestic and external funding
Creating and sustaining resources	- Availability of advanced research infrastructure - Growing international research networks and collaborations - Recognition of the need for context-specific research agendas	- Mismatch between infrastructure availability and human resources capacity - Inequities in access to research infrastructure
Producing and using research	- Strong emphasis on output-driven research (prototypes, publications, patents) - Formal establishment of institutions for evidence use, including the Health Technology Assessment Committee and the National Health Development Policy Agency (BKPK)	- Research production does not incorporate the implementation process. - Weak institutionalised pathways for research translation to policy and practice - Limited data integration and sharing across institutions.

### Governance

The governance of health research in Indonesia is shaped by a combination of policies, legal frameworks, and institutional arrangements intended to align research activities with national health priorities. While Indonesia has an established national health policy articulated in the national development plans and is planned to be operationalised through the Health Sector Master Plan *(Rencana Induk Bidang Kesehatan,* RIBK), an explicit and integrated national health research policy has not yet been formalised. As mentioned by a participant (N04): *“To identify which health topics or sectors could potentially be collaborated on with universities, we encourage academics to refer to existing strategic documents, such as the RPJMN and the Health Sector Master Plan (RIBK). We expect these documents to serve as a reference for academics to identify what roles and research topics can be pursued in collaboration with the government.”*

As one informant (N02) explained, *“The Ministry of Health is currently developing RIBK, which is derived from the health sector medium-term plan. This document identifies the indicators and activities required to achieve the targets and is expected to serve as a reference for academics in identifying potential roles and research topics*.” While RIBK provides an important reference, its role is primarily indirect because it targets the health sector as a whole rather than a national health research plan.

Health research governance is further supported by several legal and regulatory frameworks, including the Health Law, Government Regulation No. 28 of 2024, and the Ministry of Health Regulation on Health Research and Development Ethics Committees. Collectively, these regulations established ethical standards and institutional responsibilities, along with the formal basis for research governance.

The absence of a dedicated and integrated research policy has resulted in limited complementarity between health and research agendas at the national level. This is reflected in the fragmented coordination across multiple research institutions which have their own research priorities. Another challenge in the governance domain is the limited coordination between national and subnational levels. The current Indonesian NHRS plan remains highly centralised, with agenda-setting dominated by top-down mechanisms derived from national medium- and long-term development plans (N04, N03) *“There’s no single body to coordinate or monitor how all these research institutions work together (N03).”*

### Financing

Indonesia’s health research financing is primarily sourced from the state budget, distributed through BRIN and Kemendikti-Saintek. BRIN manages eight research funding schemes and submits proposals to Bappenas as part of the national planning cycle. Each institution, such as BRIN, Kemendikti-Saintek, and Kemkes, operates its own funding mechanisms. As one participant noted (N01), *“Government research funding is actually spread across many institutions, such as higher education, BRIN, LPDP, and even industry. However, these allocations tend to operate separately, without integrated coordination. They all run on their own.”*

Moreover, the total funding for health research remains low, forcing many researchers to seek external sources, such as international donors (*e.g.*, e-Asia, International Atomic Energy Agency (IAEA), and WHO), which are highly competitive and limited in scope. Participants also mentioned having to use out-of-pocket funding for publication due to a limited budget. One participant (N03) described, *“Basic research needs publication fees or conference attendance, which are often not covered. Researchers end up using their own funds or university funds, even though publications are used as performance indicators.”*

Further compounding the issue, BRIN’s grant requirements often mandate collaboration with BRIN-affiliated researchers, reducing inclusivity and discouraging independent or locally driven research. Although dissemination efforts to promote funding schemes have increased, other participants emphasised persistent funding constraints and delayed access to grant information, particularly in peripheral regions. As one respondent (W01) noted, *“Limited funding strongly affects our ability to conduct high-quality research. Important information often reaches us late, and sometimes we only learn about grant competitions after they have already closed.”*

### Creating and sustaining resources

Indonesia has made significant investments in research infrastructure, such as the availability of PET scan facilities and non-human primate laboratories under BRIN. While these facilities represent high-end research capacity, access remains limited, primarily to researchers within BRIN-affiliated institutions. Collaboration with BRIN is a prerequisite for grant eligibility and infrastructure access. External researchers need to collaborate with BRIN scientists to access their advanced facilities, which pose logistical and bureaucratic hurdles.

Furthermore, participants also expressed the mismatch between the availability of research infrastructure and expertise. BRIN has conducted outreach to universities regarding its research grant schemes, as well as non-financial support, including access to research equipment, talent management programmes and knowledge networks to address capacity gaps. One participant (N03) mentioned, *“BRIN does conduct intensive outreach activities. As heads of research organisations, we also help disseminate information about available funding schemes, as well as non‑funding programmes such as talent management, scholarships, and related opportunities.”* BRIN has also introduced seven thematic health research centres under its coordination.

Resource sharing across institutions is often constrained by unclear resource sharing mechanisms, limited interoperability between research management systems, and siloed data infrastructures. Many researchers, particularly those based outside Java Island, reported limited awareness of the existing outreach programmes. As one participant (W02) said, *“Limited funding is one of the factors that affects our ability to conduct high‑quality and productive research in* *Aceh, especially since we are located at the westernmost edge of the country. This has been a recurring challenge for us, partly because we are located at the edge of Sumatra and our alumni network is still limited.”* Importantly, BRIN informants acknowledged the information gaps, recognising that outreach mechanisms may not sufficiently reach all institutional levels.

Some participants also highlighted structural and infrastructural constraints that further limited their research capacity, noting that even when equipment was available, it was often unusable due to inadequate infrastructure and supporting facilities. One participant (E01) stated, *“Basic research tools like PCR and electrophoresis are very challenging for us to use. Sometimes the equipment exists, but the electricity capacity is insufficient, or reagents are difficult to procure.”*

### Producing and utilising research

Current Indonesian health research institutions emphasise output-driven research through publications, patents, technology readiness levels (TRL), and prototype development, particularly within universities and national research institutions. These outputs are recognised as indicators of research productivity. Although universities produce a substantial volume of health research, their work is rarely integrated into national policy processes. A participant described how studies often remain disconnected from decision-making: “*Many studies stay as reports. They are completed, but there is no clear pathway for them to enter the policy process.” (N01)* Participants argue that this may also be influenced by the limited participation of policymakers and knowledge implementers in the production phase of research.

Indonesia has shown progress in strengthening formal institutions and national surveys to support research production and utilisation. Health Technology Assessment (HTA) is perceived as a promising platform for research translation for BRIN, universities, industries, and hospitals. The HTA Committee was established to improve the uptake of research products into the health system by formulating recommendations, especially for the national health insurance. The MoH also has several large-scale research initiatives to inform national health planning and policy formulation, including Basic Health Research *(Riset Kesehatan Dasar,* Riskesdas), the Health Research Survey *(Survei Kesehatan Indonesia*, SKI), and the Indonesian Nutritional Status Survey (*Survei Status Gizi Indonesia*, SSGI). The SKI integrates Riskesdas and SSGI to assess health development achievements over the past five years and monitor trends in child nutrition, producing data representative from national to district levels. Other formal institutions were also established to bridge clinical application and policy translation, such as the Indonesian Clinical Research Unit and Health Development Agency. However one informant mentioned about the challenge of translating research into policy, “*Unfortunately, there are many limitations to the access for research outputs. There are many research outputs, but we don’t know how to access them for HTA and research translation*.” (N05).

## Discussion

This study assessed the current state of Indonesia’s NHRS and identified several challenges across governance, financing, creating and sustaining resources and producing and using research. Overall, the system remains fragmented, despite the presence of formal structures and institutions. Governance is characterised by a highly centralised, top-down approach to agenda setting, with limited involvement of local institutions. Financing relies predominantly on programmatic and proposal-driven funding mechanisms aligned with national priorities. However, it is constrained by limited sustainable domestic funding and inequitable funding access. In terms of creation and sustaining resources, the availability of advanced research infrastructure and growing international collaboration demonstrate national commitment to research, yet mismatches between infrastructure and resource capacity persist. Finally, while research production and utilisation show a strong emphasis on measurable outputs and have established formal institutions for evidence use and pathways for translating research into policy and practice remain limited.

Indonesia’s NHRS is characterised by a highly centralised, top-down agenda setting, combined with fragmented coordination across multiple research institutions. The “Consultation Background Paper” by the ESSENCE Working Group on Research Investments (WGRI), supported by WHO [22], highlights key challenges in coordinating clinical research investments in LMICs, including institutional fragmentation and unclear leadership. This contributes to the donor-driven priorities [23].

In 2022, Indonesia restructured its research governance to a single, centralised research agency (BRIN). The merger of multiple research agencies into a single national agency reflects an effort to improve coordination, efficiency, and accountability by concentrating research functions and budgets within one institution [13]. Such centralisation is not common, as several countries have established centralised agencies to enhance governance and strategic alignment in their health research systems [24,25]. However, findings from this study suggest that centralisation in Indonesia has not yet translated into system-wide coordination.

This underscores the multifaceted nature of BRIN’s role and functions. As stipulated in Presidential Regulation No. 78 of 2021, BRIN is mandated to conduct integrated research, development, assessment, and implementation activities, as well as invention and innovation [26]. Under this regulation, BRIN is assigned an overarching role within Indonesia’s research and innovation system, including regulating and formulating national research policies, providing science‑based advice to the government, strengthening science and technology human resources, facilitating and managing national research infrastructure, acting as a funding agency for research, supervising regional and local research agencies (BRIDA), and directly executing research activities [13]. The role of BRIN reflects what participants mentioned during the expert panel meeting, where BRIN serves a triple role as a funder, implementer and policy advisor.

A key consequence of the current governance arrangement is the constrained role of universities and local research institutions in shaping national research priorities with local relevance [27]. While the health sector master plan provides an important guide for health research, it functions primarily as a top-down mechanism. Currently, universities and local research institutions serve as implementers of the agenda rather than actively participating in priority setting for health research. The absence of a co-creation mechanism may hinder the role of research in addressing health needs at the local level [11]. One scoping review concludes that successful governance is consistently characterised by clearly defined cross‑governmental and institutional mandates, coordinated leadership, and formal mechanisms that promote collaboration across health and allied sectors [28].

The centralised research system in Indonesia contrasts with Thailand’s more pluralistic governance model [29]. While Thailand’s decentralised approach has enabled flexibility, innovation, and stronger stakeholder engagement, particularly through participatory mechanisms, the approach has also been constrained by fragmented coordination and limited system‑level stewardship due to the absence of an actor responsible for aligning priorities and resources across institutions. Effective health research governance depends less on the degree of centralisation itself than on clear role delineation, institutionalised coordination mechanisms, and inclusive priority‑setting processes that balance national strategic direction with responsiveness to local health needs [2]. Taken together, these cases illustrate that both centralised and decentralised NHRS governance models involve trade‑offs. In Indonesia, a similar decentralised effort is underway through the formation of BRIDA to enhance the responsiveness of innovation for local development needs [30]. As a subnational entity operating within BRIN’s framework, BRIDA has the potential to strengthen vertical coordination and facilitate local relevance with national health priorities.

Coordination between research organisations in Indonesia requires clear roles and strategic alignment. Strengthening subnational platforms and standardising monitoring can bridge national–subnational gaps and enhance the impact of Indonesia’s health research system. Consistent with findings from the London School of Economics (2021) study in Africa [31], which emphasised the need for research leadership and alignment of priorities, this study illustrates how gaps in research governance and coordination may hinder health research financing, management, and utilisation, particularly at the subnational and local levels [29].

A comparative NHRS analysis using the WHO Framework in an Asian context remains scarce, compared to the African region. However, a similar finding from Mauritius reported that the weakest performance of its NHRS was in leadership and governance, driven by the absence of national research for health policy and a prioritised research agenda [15]. Similar challenges have been documented in Thailand, where assessments of the national health research system identified weak leadership and the absence of a coordinating body to steer the system [29]. In the Thai context, multiple organisations engage in health research, each with its own priority setting. approaches.

In terms of financing, the Global Consultative Expert Working Group on Research and Development: Financing and Coordination has recommended that governments commit at least 0.2% of GDP to health research and 0.01% to research on neglected diseases in developing countries [32]. Unfortunately, the proportion of Indonesia’s health R&D expenditure to its GDP is still unknown and not reported in the WHO GERD report [10]. This lack of data makes it difficult to understand the country’s health research capacity and compare the capacity within the region. By contrast, neighbouring countries such as Vietnam (0.01%), Malaysia (0.09%), and Singapore (0.43%) have their data on health R&D relative to GDP available [10]. In 2020, the Indonesia MoH allocated more than US$37 million to its Health Research and Development Agency, but total funding remains hard to trace due to fragmented distribution across agencies [16]. Based on the latest report, the total budget of BRIN is approximately US$84 million across nine priority areas. However, data regarding specific BRIN’s budget for health research remains scarce [16].

Despite these national allocations, domestic funding remains insufficient to meet the scale and diversity of health research needs, which leads to reliance on external donor funding as commonly found in LMICs [6,7]. Donor resources contribute important financial and technical inputs, particularly in areas where domestic funding is constrained. However, donor-funded research is often driven by global funders’ priorities rather than national or subnational health needs [4]. As a result, while donor support expands research activity, it may not directly translate to strengthening national research agendas or institutional capacity in the long term [33].

There is also an absence of a unified national framework to guide and align both domestic and external investments. Without such a framework, funding, whether domestic or donor-supported, risks reinforcing fragmentation rather than building equitable research capacity [34]. High-income countries such as Finland and the UK, their R&D roadmaps are structured with clearly outlined investment targets, strategic pillars, and implementation mechanisms [35,36].

Access to information on funding opportunities is also uneven, particularly for universities and research centres outside major research hubs. Although BRIN has introduced competitive calls for proposals to widen funding opportunities, findings from this study suggest that information often reaches local researchers too late, and it reinforces existing inequities in access to research funding. Similar challenges have been observed in Kenya, where rural and peripheral regions continue to have limited reach to health research funding [37].

Indonesia has demonstrated commitment to advancing health research by investing in advanced laboratory facilities, reagents, whole-genome sequencing, and the establishment of a formal institution for clinical research. Furthermore, the findings indicate growing international research networks and collaborations, partly driven by funding requirements that encourage partnerships with international researchers. Despite these advances, participants expressed a mismatch between the availability of research infrastructure and appropriate human expertise**.** There is limited information about facilities mapping across research institutions. Moreover, resource sharing across institutions remains constrained by limited socialisation and logistical issues.

In addition to institutional barriers, technical problems are also hindering the utilisation of available resources. Participants reported that even when equipment was available, the infrastructure was often inadequate, including electricity availability and procurement. As a result, the full potential of Indonesia’s research ecosystem remains underutilised, especially outside major urban research hubs. Consistent with previous literature, Indonesia’s current situation illustrates that investments in research infrastructure do not always translate into improvement in research capacity without mechanisms linking expertise, access, and facilities [14,38,39]. Taken together, the findings suggest that a national research database and infrastructure mapping is, therefore, important to facilitate collaboration and resource management across institutions.

Furthermore, Indonesia has established multiple institutions for translating and communicating research to inform health policy and practice, including the HTA committee and BKPK. The challenge, therefore, is not institutional absence but rather institutional coordination for research utilisation. The HTA committee represents a formal mechanism through which evidence can be used to inform decisions. However, findings from this study suggest that health research is commonly produced with limited involvement of policymakers and users. Producing and using research remains largely conducted in silos within each institution, with limited integration into the broader NHRS. The available data mainly serves as part of the health services, not purposefully designed for research and HTA.

The separation between knowledge production, utilisation, and translation observed in Indonesia may partly explain why academic research remains disconnected from policy and practice. A prior qualitative study conducted across six LMICs academic institutions, including Indonesia, identified the institutional drivers of knowledge translation, including the complexity of policy processes, the need for soft skills to engage with policymakers and misalignment between institutional missions and incentives for knowledge translation [11]. Key recommendations to strengthen research uptake for health policymaking in LMICs include establishing evidence translation platforms, improving policy research literacy, and accounting for political context [40].

A systematic review on barriers to evidence use by policymakers reported that the most frequent barriers were poor access to high-quality relevant research and a lack of timely research output, while the most consistent facilitator was collaboration between researchers and policymakers [41]. The poor access to high-quality, relevant and timely research outputs was expressed by participants in this study. In Indonesia, however, formal mechanisms for research co-production remain limited. The current situation contrasts with settings where research co-production is institutionalised, such as in the UK, where priority-setting mechanisms involving researchers, communities, and policymakers are embedded within the National Institute for Health and Care Research [42,43].

Publication of peer-reviewed articles in journals is one of the main methods to disseminate research [14]. Indonesian health research productivity prioritises output metrics like peer-reviewed publications, TRL, and prototypes. Although these metrics offer a clear indicator, they may shift the focus towards numbers instead of relevance to policy and practice. The “publish or perish” culture exacerbates this [44], as publication counts serve as key performance indicators despite limited funding, forcing researchers to cover out-of-pocket costs for journals or conferences.

While Indonesia demonstrates strong capacity for generating data, pathways for the utilisation of evidence across institutions remain underdeveloped. The MoH has invested various research efforts in the form of national-level health surveys, which generate extensive data, yet these data are not optimised for use across institutions. Limited accessibility may hinder their use for broader health research purposes. Thus, it calls for a unified national research plan along with concrete pathways to translate research findings into policy and practice. Strengthening the linkage between producing and utilising research is crucial to improve the benefits of health research at the population level.

### Strengths

This paper contributes to the limited literature on the NHRS in low and middle-income countries. By examining the Indonesian context, it may reflect similar NHRS challenges identified in other LMIC settings. This study proposes context-specific recommendations, including the need for a unified research roadmap, coordination mechanisms, and strengthened subnational research bodies to bridge national strategies with local needs. Further research across other countries is essential to inform NHRS development and encourage context-based reforms in similar settings.

### Limitations

The findings of this study should be interpreted cautiously with the following limitations. First, variations in governance and decision-making structures among institutions involved in health research in our context may influence the interpretation and applicability of our findings. Second, data collection was conducted in a hybrid format, which may have affected the dynamics of interaction and limited equal participation opportunities between in-person and virtual attendees. Third, the findings are contextual in our setting, which limits the generalisability. Given these limitations and the complexity of policy and institutional contexts, further research to explore the NHRS in other LMICs is necessary to support policy recommendations.

## Conclusion

Indonesia’s health research system has made structural progress, yet coordination and implementation remain fragmented, shaped by weak governance, constrained financing, unequal access to resources and limited translation to policy and practice. Addressing these gaps requires stronger inter-institutional coordination, dedicated funding mechanisms, investment in research capacity, and coherent national implementation policies. The study findings are, however, constrained by its reliance on document analysis and a limited range of stakeholder perspectives, which may not fully reflect realities at the subnational level. Future research employing multi-level analysis and mixed-methods approaches is needed to better understand the challenges of strengthening Indonesia’s NHRS.

## Supporting information

S1 File Appendix 1. Questionnaire on Country Resources for Health Research. (DOC)

## Abbreviations

APBN: Anggaran Pendapatan dan Belanja Negara (National State Budget)
Balitbangkes: Badan Penelitian dan Pengembangan Kesehatan (National Institute of Health Research and Development)
BKPK-Kemenkes: Badan Kebijakan Pembangunan Kesehatan, Kementerian Kesehatan (Health Development Policy Agency, Ministry of Health)
BRIDA: Deputi Bidang Riset dan Inovasi Daerah (Deputy for Regional Research and Innovation)
BRIN: Badan Riset dan Inovasi Nasional (National Research and Innovation Agency)
CRO: Contract Research Organization
CRU: Clinical Research Unit
DIPA: Daftar Isian Pelaksanaan Anggaran (Budget Implementation List)
GERD: Gross Domestic Research and Development Expenditure on Health
GDP: Gross Domestic Product
HICs: High-Income Countries
HRS: Health Research System
HTA-Kemenkes: Health Technology Assessment Division, Ministry of Health
ICMR: Indian Council of Medical Research
INA-CRC: Indonesia Clinical Research Center
KEMRI: Kenya Medical Research Institute
Kemendikti-Saintek: Kementerian Pendidikan Tinggi, Sains, dan Teknologi
Kemkes: Kementerian Kesehatan (Ministry of Health)
Kemenag: Kementerian Agama (Ministry of Religious Affairs)
KII: Key Informant Interview
LIPI: Lembaga Ilmu Pengetahuan Indonesia
LMICs: Low- and Middle-Income Countries
LPPM: Lembaga Penelitian dan Pengabdian kepada Masyarakat (Research and Community Service Institutions)
MLP: Medium- and Long-Term Plan
NHRS: National Health Research System
NHRMF: National Health Research Management Forum
NIHR: National Institute for Health Research
OR Kesehatan, BRIN: Organisasi Riset Kesehatan, Badan Riset dan Inovasi Nasional (Health Research Organisation of the National Research and Innovation Agency)
Renstra: Rencana Strategis (Strategic Plan)
Riskesdas: Riset Kesehatan Dasar (Basic Health Research)
RIBK: Rencana Induk Bidang Kesehatan (Health Sector Master Plan)
RMRCs: Regional Medical Research Centres
RPJMN: Rencana Pembangunan Jangka Menengah Nasional (National Medium-Term Development Plan)
SCAT: Steps for Coding and Theorization
SKI: Survei Kesehatan Indonesia (Health Research Survey)
SKP: Sasaran Kinerja Pegawai (Work Target Agreement)
SSGI: Survei Status Gizi Indonesia (Indonesian Nutritional Status Survey)
TRL: Technology Readiness Levels
WHO: World Health Organization

## References

[1] Nass SJ, Levit LA, Gostin LO. Beyond the HIPAA privacy rule: enhancing privacy, improving health through research. Nass SJ, Levit LA, Gostin LO, editors. Washington, D.C.: National Academies Press; 2009. doi: 10.17226/12458 20662116

[2] World Health Organization. National health research systems. Cha-am, Thailand: World Health Organization; 2001. https://iris.who.int/server/api/core/bitstreams/e2c2447b-ea40-4262-83d0-c10d62e643cd/content

[3] Hanney SR, Kanya L, Pokhrel S, Jones TH, Boaz A. How to strengthen a health research system: WHO’s review, whose literature and who is providing leadership?. Health Res Policy Syst. 2020;18(1):72. doi: 10.1186/s12961-020-00581-1 32571364 PMC7308111

[4] McGregor S, Henderson KJ, Kaldor JM. How are health research priorities set in low and middle income countries? A systematic review of published reports. PLoS One. 2014;9(9):e108787. doi: 10.1371/journal.pone.0108787 25275315 PMC4183511

[5] Hasan BS, Rasheed MA, Wahid A, Kumar RK, Zuhlke L. Generating evidence from contextual clinical research in low- to middle income countries: a roadmap based on theory of change. Front Pediatr. 2021;9:764239. doi: 10.3389/fped.2021.764239 34956976 PMC8696471

[6] Alliance for Health Policy and Systems Research. Analyzing donor funding flows in support of health policy and systems research in low- and middle-income countries from 2000-14. https://wkc.who.int/docs/librariesprovider11/publications/supplementary-material/alliancehpsr_fundflowshpsr.pdf?sfvrsn=8f061cc7_7

[7] Grépin KA, Pinkstaff CB, Shroff ZC, Ghaffar A. Donor funding health policy and systems research in low- and middle-income countries: how much, from where and to whom. Health Res Policy Syst. 2017;15:68. doi: 10.1186/s12961-017-0224-6 28854946 PMC5577666

[8] Cordero C, Delino R, Jeyaseelan L, Lansang MA, Lozano JM, Kumar S, et al. Funding agencies in low- and middle-income countries: support for knowledge translation. Bull World Health Organ. 2008;86(7):524–34. doi: 10.2471/blt.07.040386 18670664 PMC2647493

[9] Ravi K, Bentounsi Z, Tariq A, Brazeal A, Daudu D, Back F, et al. Systematic analysis of authorship demographics in global surgery. BMJ Glob Health. 2021;6(10):e006672. doi: 10.1136/bmjgh-2021-006672 34666988 PMC8527109

[10] WHO. Gross domestic R&D expenditure on health (health GERD) as a % of GDP. Accessed 2025 December 19. https://www.who.int/observatories/global-observatory-on-health-research-and-development/indicators/gross-domestic-r-d-expenditure-on-health-as-a-percent-of-gross-domestic-product

[11] Kalbarczyk A, Rodriguez DC, Mahendradhata Y, Sarker M, Seme A, Majumdar P, et al. Barriers and facilitators to knowledge translation activities within academic institutions in low- and middle-income countries. Health Policy Plan. 2021;36(5):728–39. doi: 10.1093/heapol/czaa188 33661285 PMC8173595

[12] Amelia IR, Jannah LM. Analysis of national research and innovation agency (BRIN) transformation readiness to accelerate national research and innovation development using swot analysis. DiA. 2022;20(02):331–50. doi: 10.30996/dia.v20i02.7056

[13] Burhani AN. Governing research in Indonesia: present and future challenges. 1st ed. Singapore: ISEAS - Yusof Ishak Institute; 2026.

[14] Kirigia JM, Ota MO, Motari M, Bataringaya JE, Mouhouelo P. National health research systems in the WHO African Region: current status and the way forward. Health Res Policy Syst. 2015;13:61. doi: 10.1186/s12961-015-0054-3 26519052 PMC4628337

[15] Musango L, Nundoochan A, Ramful Y, Kirigia JM. An assessment of the performance of the national health research system in Mauritius. BMC Health Serv Res. 2023;23(1):218. doi: 10.1186/s12913-023-09208-x 36879247 PMC9990251

[16] Indonesia. Alliance for HPSR. Accessed 2025 October 25. https://www.ahpsr.org/partners-hpsr-report/country-profiles/indonesia/

[17] Archibald MM, Ambagtsheer RC, Casey MG, Lawless M. Using zoom videoconferencing for qualitative data collection: perceptions and experiences of researchers and participants. Int J Qualit Methods. 2019;18. doi: 10.1177/1609406919874596

[18] Gale NK, Heath G, Cameron E, Rashid S, Redwood S. Using the framework method for the analysis of qualitative data in multi-disciplinary health research. BMC Med Res Methodol. 2013;13:117. doi: 10.1186/1471-2288-13-117 24047204 PMC3848812

[19] Pang T, Sadana R, Hanney S, Bhutta ZA, Hyder AA, Simon J. Knowledge for better health: a conceptual framework and foundation for health research systems. Bull World Health Organ. 2003;81(11):815–20. 14758408 PMC2572351

[20] Otani OT. ‘SCAT’ a qualitative data analysis method by four-step coding: easy startable and small scale data-applicable process of theorization. 2007. Accessed 2025 October 25. https://www.semanticscholar.org/paper/%22SCAT%22-a-qualitative-data-analysis-method-by-easy-%E5%A4%A7%E8%B0%B7-Otani/84c6fe6c730b378ac8f651e193ca2cf1467ea74a

[21] Kirigia JM, Wambebe C. Status of national health research systems in ten countries of the WHO African Region. BMC Health Serv Res. 2006;6:135. doi: 10.1186/1472-6963-6-135 17052326 PMC1622748

[22] Eigbike M. Health research capacity strengthening in low and middle-income countries: current situation and opportunities to leverage data for better coordination and greater impact. 2020. https://tdr.who.int/docs/librariesprovider10/essence/essence-mechanism-consultant-report-2020.pdf

[23] Fosci M, Loffreda L, Velten L, Johnson R. Research capacity strengthening in LMICs. The UK Department for International Development; 2019. https://assets.publishing.service.gov.uk/media/5d42be4eed915d09d8945db9/SRIA_-_REA_final__Dec_2019_Heart___003_.pdf

[24] National Council for Law Reporting. The Kenya medical research institute order. National Council for Law Reporting; 2021.

[25] Sun Y, Cao C. Planning for science: China’s “grand experiment” and global implications. Humanit Soc Sci Commun. 2021;8:215. doi: 10.1057/s41599-021-00895-7

[26] President of Republic Indonesia. Perpres No. 78 Tahun 2021. 2021. http://peraturan.bpk.go.id/Details/178084/perpres-no-78-tahun-2021

[27] Juma PA, Jones CM, Mijumbi-Deve R, Wenham C, Masupe T, Sobngwi-Tambekou J, et al. Governance of health research in four eastern and southern African countries. Health Res Policy Syst. 2021;19(1):132. doi: 10.1186/s12961-021-00781-3 34645454 PMC8513324

[28] Saikat S, Seifeldin R, Zhang Y, Nweje M, Shivji S, Schmets G, et al. Governance for public health across health and allied sectors: a scoping review. BMJ Public Health. 2025;3(2):e003542. doi: 10.1136/bmjph-2025-003542 41211583 PMC12593447

[29] Jongudomsuk P. Health research system for sustainable health reform in Thailand. Minist Public Health Thail. 2008;2.

[30] Saksono H. Urgensi pembentukan dan posisi strategisnya sebagai solusi permasalahan pembangunan dan peningkatan kinerja pemerintahan daerah. Nakhoda J Ilmu Pemerintah. 2021;20:178–96. doi: 10.35967/njip.v20i2.290

[31] Jones CM, Ankotche A, Canner E, Habboubi F, Mamuye H, Hedquist A, et al. Strengthening national health research systems in Africa: lessons and insights from across the continent. Wellcome Trust. 2021. doi: 10.6084/M9.FIGSHARE.14039807

[32] World Health Organization. Consultative expert working group on research and development: financing and coordination. World Health Organization; 2012. https://apps.who.int/gb/cewg/pdf_files/A65_24-en.pdf

[33] Charani E, Abimbola S, Pai M, Adeyi O, Mendelson M, Laxminarayan R. Funders: the missing link in equitable global health research?. PLOS Glob Public Health. 2022;2:e0000583. doi: 10.1371/journal.pgph.0000583 36962429 PMC10021882

[34] Fadlallah R, El-Jardali F, Chidiac N, Daher N, Harb A. Analysis of funding landscape for health policy and systems research in the Eastern Mediterranean Region: a scoping review of the literature over the past decade. Health Res Policy Syst. 2024;22(1):70. doi: 10.1186/s12961-024-01161-3 38915031 PMC11194879

[35] Ministry of Education and Culture F. The National roadmap for research, development and innovation. 2021. https://okm.fi/documents/1410845/22508665/UpdatedRDIRoadmap2021.pdf/2ddb19a7-0e2e-a24f-69b8-51638dcaea02/UpdatedRDIRoadmap2021.pdf?t=1642765048947

[36] Parry A. UK research and development roadmap. The National Archives; 2020.

[37] Nesidai BA, Kithuka PM, Mekala EK, Njoroge PW, Karanja PM. Mapping health research capacity building initiatives in Kenya: a scoping review. East Afr J Health Sci. 2024;8:270–83. doi: 10.37284/eajhs.8.2.3517

[38] Vasquez EE, Hirsch JS, Giang LM, Parker RG. Rethinking health research capacity strengthening. Glob Public Health. 2013;8 Suppl 1(0 1):S104-24. doi: 10.1080/17441692.2013.786117 23651463 PMC3778121

[39] Kengia JT, Kalolo A, Barash D, Chwa C, Hayirli TC, Kapologwe NA, et al. Research capacity, motivators and barriers to conducting research among healthcare providers in Tanzania’s public health system: a mixed methods study. Hum Resour Health. 2023;21(1):73. doi: 10.1186/s12960-023-00858-w 37670321 PMC10478476

[40] Schleiff MJ, Kuan A, Ghaffar A. Comparative analysis of country-level enablers, barriers and recommendations to strengthen institutional capacity for evidence uptake in decision-making. Health Res Policy Syst. 2020;18(1):78. doi: 10.1186/s12961-020-00546-4 32646439 PMC7350720

[41] Oliver K, Innvar S, Lorenc T, Woodman J, Thomas J. A systematic review of barriers to and facilitators of the use of evidence by policymakers. BMC Health Serv Res. 2014;14:2. doi: 10.1186/1472-6963-14-2 24383766 PMC3909454

[42] Crocker JC, Moore L, Ogden M, Crowe S, Khan M, Schoemaker C, et al. Overarching priorities for health and care research in the United Kingdom: a coproduced synthesis of james lind alliance “Top 10s”. Health Expect. 2024;27(3):e14096. doi: 10.1111/hex.14096 38895996 PMC11187853

[43] NIHR. Who we are - National institute for health and care research. Accessed 2025 December 25. https://www.nihr.ac.uk/about-us/who-we-are

[44] Grech V. Publish or perish, information overload, and journal impact factors - A conflicting tripod of forces. Saudi J Anaesth. 2022;16(2):204–7. doi: 10.4103/sja.sja_632_21 35431753 PMC9009569

