# Supplementary material for: Assessing National Health Research System in a resource-limited setting: Insights from Indonesia
Source: PLoS One. 2026 Jun 4;21(6):e0350393. doi: 10.1371/journal.pone.0350393 (PMC13235909; doi:10.1371/journal.pone.0350393)
Supplement: S1 File — (DOC) [file pone.0350393.s001.doc]

# Appendix 1: Questionnaire on Country Resources for Health Research

**Country name: _________________________________________**

Name:

Address:

Telephone:

Fax:

Email:

**Questionnaire administrator: ______________________________**

**Filled in: Month ______________ Year ____________________**

***1. Health Research Policy***

- 1. Does the country have an official national health policy (NHP)? 1=Yes, 0=No. 
  2. Does the country have a strategic health plan (SHP)? 1=Yes, 0=No. 
  3. Does the country have an official health research policy (HRP)? 1=Yes, 0=No. 
  4. Please indicate the year of its initial formulation.    
  5. Does the health research policy document have:

1. Preamble? 1=Yes, 0=No. 
2. A situation analysis of health research in the country? 1=Yes, 0=No. 
3. A strategic vision for health research in the country (including vision, goals, underlying values, guiding principles, research priorities, implementation strategies, resource mobilization mechanisms, modalities for monitoring and evaluation, etc)? 1=Yes, 0=No. 
4. An organizational or working plan of the national health research system? 1=Yes, 0=No. 
5. Strategic vision for the assessment of the system? 1=Yes, 0=No. 
6. The national health research policy statement (aims, objectives)? 1=Yes, 0=No. 
7. Any other (specify)____________________________________

______________________________________________________

- 1. If the answers in 1.1 and 1.3 are ‘YES’, is there complementarity between the national health policy and the health research policy? 1=Yes, 0=No. 
  2. If the answer in 1.3 is ‘YES’, were the following involved in the process of formulating the HRP:

(a) Universities – especially faculties of health sciences? 1=Yes, 0=No. 

1. Medical research councils or institutes? 1=Yes, 0=No. 
2. Representatives of non-governmental hospitals? 1=Yes, 0=No. 
3. Provincial (or regional) medical officers of health? 1=Yes, 0=No. 
4. District medical officers of health? 1=Yes, 0=No. 
5. National medical association(s)? 1=Yes, 0=No. 
6. Administrators of health research policy? 1=Yes, 0=No. 
   1. If health research policy already exists, does it need to be updated? 1=Yes, 0=No. 
   2. If health research policy does not presently exist, are the policy-makers interested in developing it? 1=Yes, 0=No. 
   3. If “YES” in 1.9, will there be need for support from WHO in developing the health research policy? 1=Yes, 0=No. 
   4. If “Yes” in 1.10, what form of support would be needed from WHO?
   5. Technical support to draft the health research policy? 1=Yes, 0=No. 
   6. Guidelines on formulation of national health research policy?

1=Yes, 0=No. 

- 1. Financial support for undertaking a health research situation analysis? 1=Yes, 0=No. 
  2. Technical guidance in the development of grant proposals? 1=Yes, 0=No. 
  3. Human capacity development for implementation of health research policy? 1=Yes, 0=No. 
  4. Sharing of experiences and lessons from countries with health research policy? 1=Yes, 0=No. 

*Please attach a copy of the health research policy document, if available.*

***2. Health Research Legislation***

2.1 Does the country have a law relating to health research? 1=Yes, 0=No. 

2.2 If “Yes” in 2.1, provide the title of the most recent law related to health research.

(a) Title:

(b) Does the law include ethical concerns? 1=Yes, 0=No. 

(c) In which year was the law enacted?    

*Please attach a copy, if available.*

***3. Strategic Health Research Plan***

- 1. Does the country have a strategic health research plan? 1=Yes, 0=No. 
  2. If “YES” in 3.1, what period does the plan cover?     to    

3.3 What is the title of the plan?

3.4 Does the plan need updating? 1=Yes, 0=No. 

3.5 If “Yes” in 3.4, do you need support from WHO to update the plan? 1=Yes, 0=No. 

3.6 Is the plan being implemented? 1=Yes; 0=No. 

*Please attach a copy, if available.*

***4. Research Coordination Mechanisms***

- 1. Is there a functional national health research system (NHRS)? 1=Yes, 0=No. 
  2. If “YES” in 4.1, does the NHRS have clear terms of reference? 1=Yes, 0=No. 

*Please attach a copy of the terms of reference.*

- 1. Is there a functional national health research management forum (NHRMF)?

1=Yes, 0=No. 

4.4 If “YES” in 4.3, does the NHRMF have clear terms of reference? 1=Yes, 0=No. 

*Please attach a copy of the terms of reference.*

- 1. Does the country have a functional ethical review committee (ERC)?

1=Yes, 0=No. 

- 1. If “YES” in 4.5, does the ERC have written terms of reference? 1=Yes, 0=No. 

*Please attach a copy of the terms of reference.*

- 1. How regularly does the ERC meet? 1=Monthly, 2=Quarterly, 3=Semi-annually,

4=Annually, 5=Whenever there are projects to review, 6=Has never met. 

- 1. Does the country have a scientific review committee (SRC)? 1=Yes, 0=No. 
  2. If “YES” in 4.8, does the SRC have written terms of reference? 1=Yes, 0=No. 

*Please attach a copy of the terms of reference.*

- 1. How regularly does the SRC meet? 1=Monthly, 2=Quarterly, 3=Semi-annually, 4=Annually, 5=Whenever there are projects to review, 6=Has never met. 
  2. Are there health institutions with institutional review committees (IRC)?

1=Yes, 0=No. 

- 1. Are there hospitals with ethical review committees to review all clinical research proposals? 1=Yes, 0=No. 
  2. Is there a national health research focal point in the country? 1=Yes, 0=No. 
  3. Are there national guidelines on development of collaboration agreements on health research involving health institutions and agencies outside the country?

1=Yes, 0=No. 

*Please attach a copy of such agreement.*

4.15 Is there a national network of health research and development which includes, among others:

1. Universities – especially faculties of health sciences? 1= Yes, 0=No. 
2. Medical research councils or institutes? 1=Yes, 0=No. 
3. Representatives of non-governmental hospitals? 1=Yes, 0=No. 
4. Provincial (or regional) medical officers of health? 1=Yes, 0=No. 
5. District medical officers of health? 1=Yes, 0=No. 
6. National medical association(s)? 1=Yes, 0=No. 

*Please attach a page with the contact addresses (postal address, telephone, fax, e-mail) of the chairpersons of the NHRS, NHRMF, ERC, IRC and SRC.*

***5. Health Research Programme***

- 1. Does the country have a health research programme (HRP)? 1=Yes, 0=No. 
  2. If “YES” in 5.1, when was the programme constituted?    
  3. Does the programme have a mission statement? 1=Yes, 0=No. 
  4. Does the programme have clearly defined terms of reference? 1=Yes, 0=No. 
  5. Does the programme have a clearly defined organizational structure?

1=Yes, 0=No. 

- 1. How many technical and support staff are there in the programme?________
  2. Does the programme have a plan of action? 1=Yes, 0=No. 
  3. How many computers does the programme have?___________________
  4. Is the programme connected to e-mail and Internet? 1=Yes, 0=No. 
  5. In what government ministry or department is the programme housed? ___________
  6. What is the annual budget of the health research programme?
  7. Does the HRP undertake research by itself? 1=Yes, 0=No. 
  8. If yes to 5.12, please list the titles of the studies that the HRP carried out last year.

*Please attach a copy of: (a) the latest plan of action, if available; (b) a list of the studies carried out by HRP in 2002, with an indication of how the findings from each were used in health policy development or health services management.*

***6. Research Institutes***

6.1 Does the country have a national health research institute (NHRI)? 1=Yes; 0=No. 

6.2 If “YES” in 6.1, please provide below the contact details of the director of the institute.

Name:__________________________________________________

Postal address:_________________________________________

Telephone number:_______________________________________

Fax number:_____________________________________________

E-mail:_________________________________________________

Website:________________________________________________

6.3 If “YES” in 6.1, when was the institute started?    

6.4 Is the NHRI under the Ministry of Health? 1=Yes, 0=No. 

6.5 If “NO” in 6.4, under which ministry is it?_____________________

6.6 If “YES” in 6.1, please attach a list of: (a) personnel in the institute (indicating their specialities); (b) research priorities; (c) published research outputs for the last three years.

- 1. Does the NHRI have: (a) telephone facilities? 1=Yes, 0=No. 

(b) fax machine(s)? 1=Yes, 0=No. 

(c) scanner(s)? 1=Yes, 0=No. 

- 1. Does each researcher in the NHRI have a computer and a printer? 1=Yes, 0=No. 
  2. Does each researcher in the NHRI have access to e-mail and Internet?

1=Yes, 0=No. 

- 1. Is there a memorandum of understanding between the Ministry of Health and the NHRI? 1=Yes, 0=No. 

*Please attach a copy of the MOU.*

- 1. Does the Ministry of Health from time to time commission the NHRI to undertake operations research? 1=Yes, 0=No. 
  2. If “YES” in 6.11, attach a list of the studies undertaken by the NHRI for the Ministry of Health use over the last two years.
  3. Is the NHRI a WHO collaborating centre? 1=Yes, 0=No. 
  4. What are the different ways that the NHRI disseminates its research?

1. (c)
2. (d)
   1. What are the five key enabling and constraining factors for health research in medical research councils or health research institutes?

*List them in the table below.*

| Key health research factors: Councils and institutes | | |
| --- | --- | --- |
| Name of institution | Enabling factors | Constraining factors |
| 1. |  |  |
| 2. |  |  |
| 3. |  |  |
| 4. |  |  |
| 5. |  |  |

***7. National Universities***

- 1. Please provide a list of national universities with faculties of health sciences and their contact details, i.e. name of the dean, postal address, telephone number, fax number, e-mail and website.
  2. Do the faculties of health sciences mentioned in 7.1 conduct research? 1=Yes, 0=No. 
  3. For those faculties of health sciences that conduct research, please attach a list of the:

1. personnel (indicating their specialities)
2. research priorities
3. published research for last three years
   1. How many researchers mentioned in 7.2 above have their own computers?_________
   2. Do the faculties of health sciences in the universities mentioned in 7.1 each have a memorandum of understanding with the Ministry of Health (MoH)? 1=Yes, 0=No. 
   3. If yes in 7.5, is the memorandum of understanding about:
4. Developing human resources for MoH? 1=Yes, 0=No. 
5. Providing technical advice to MoH? 1=Yes, 0=No. 
6. Undertaking research for MoH? 1=Yes, 0=No. 
   1. What are the five key enabling and constraining factors for health research in the universities?

*List them in the table below.*

| Key health research factors: Universities | | |
| --- | --- | --- |
| Name of university | Enabling factors | Constraining factors |
| 1. |  |  |
| 2. |  |  |
| 3. |  |  |
| 4. |  |  |
| 5. |  |  |

***8. Health Research Financing and Budget***

8.1 Is there a budget line for health research in the Ministry of Health budget document?

1=Yes, 0=No. 

8.2 How much money (in local currency) did the Ministry of Health allocate to research in 2001 and 2002?

8.3 What was the Ministry of Health’s overall budget (in local currency) in 2001 and 2002?

8.4 Please provide an estimate of the total government budgetary allocation (in local currency) to health research in 2001.

8.5 What was the total government budget in 2002 and 2003?

8.6 Approximately how much money (in local currency), from all sources, was spent on health research last year?____________________________________

8.7 How is health research financed in the country? Please rank the following in order of decreasing importance (1 for the most important method and 6 for the least)

Government tax revenues____

Private sector companies______

Multilateral and bilateral donor funding_____

Local NGOs_____

International NGOs_____

Other (specify)______

***9. Nongovernmental Organizations Involved in Health Research***

- 1. Are there any NGOs in the country that undertake health research? 1=Yes, 0=No. 

*Please attach a page with names, contact information and sources of funding for those NGOs that undertake health research.*

***10. Actions Needed to Strengthen Health Research Capacity***

10.1 Please indicate in the table below the actions that should be taken at the local and international levels to stimulate health research.

| Actions needed at country level | Actions needed at international level |
| --- | --- |
|  |  |
|  |  |
|  |  |
|  |  |
|  |  |
|  |  |
|  |  |

**Thank you for your cooperation.**
